# Supplementary material for: Single cell transcriptomic analysis reveals cellular diversity of murine esophageal epithelium
Source: Nat Commun. 2022 Apr 20;13:2167. doi: 10.1038/s41467-022-29747-x (PMC9021266; doi:10.1038/s41467-022-29747-x)
Supplement: Supplementary file 1 — Supplementary Information [file 41467_2022_29747_MOESM1_ESM.docx]

**Supplementary Information**

‘Single cell transcriptomic analysis reveals cellular diversity of murine esophageal epithelium’

MF Kabir and AL Karami, et al.


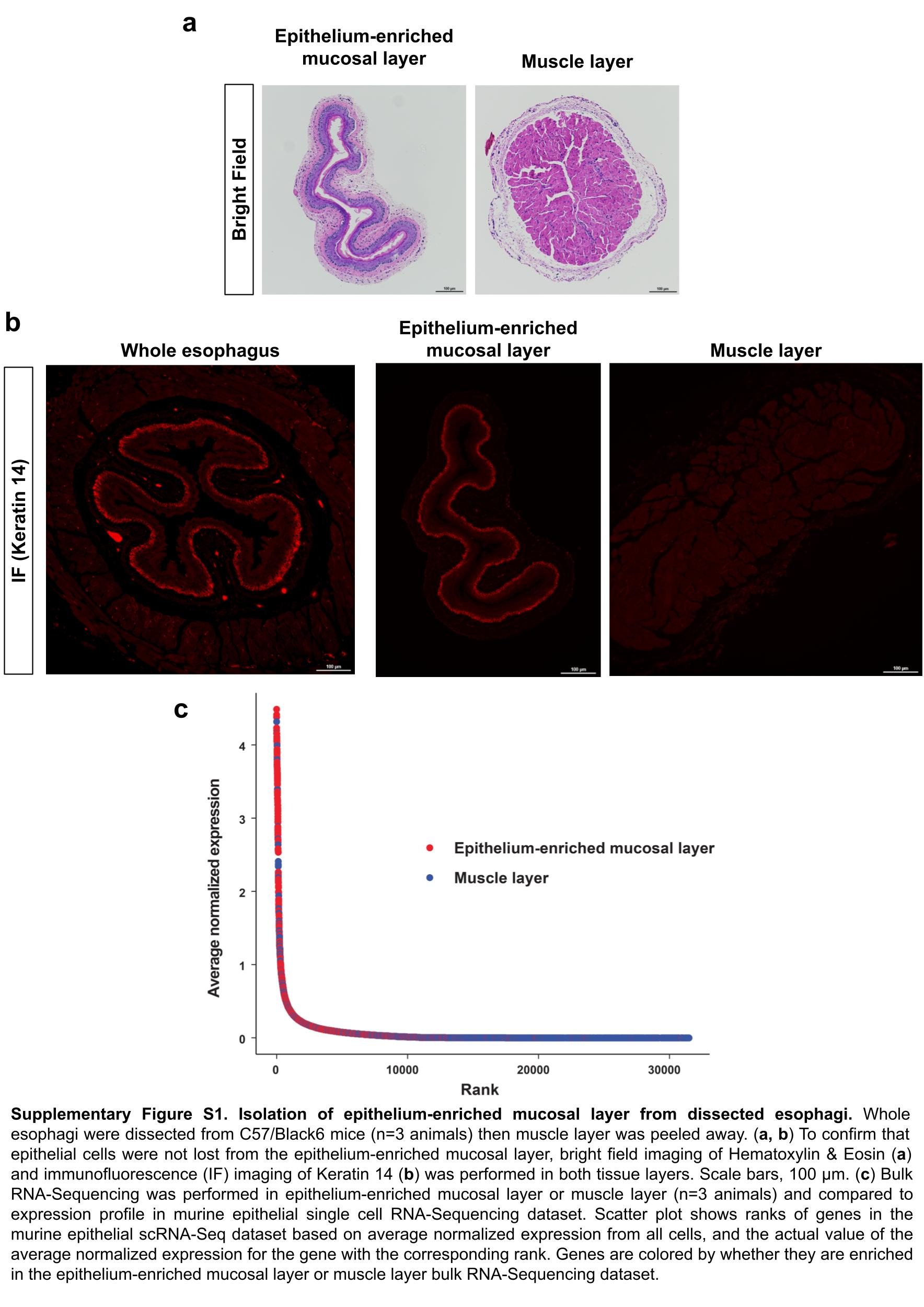


**
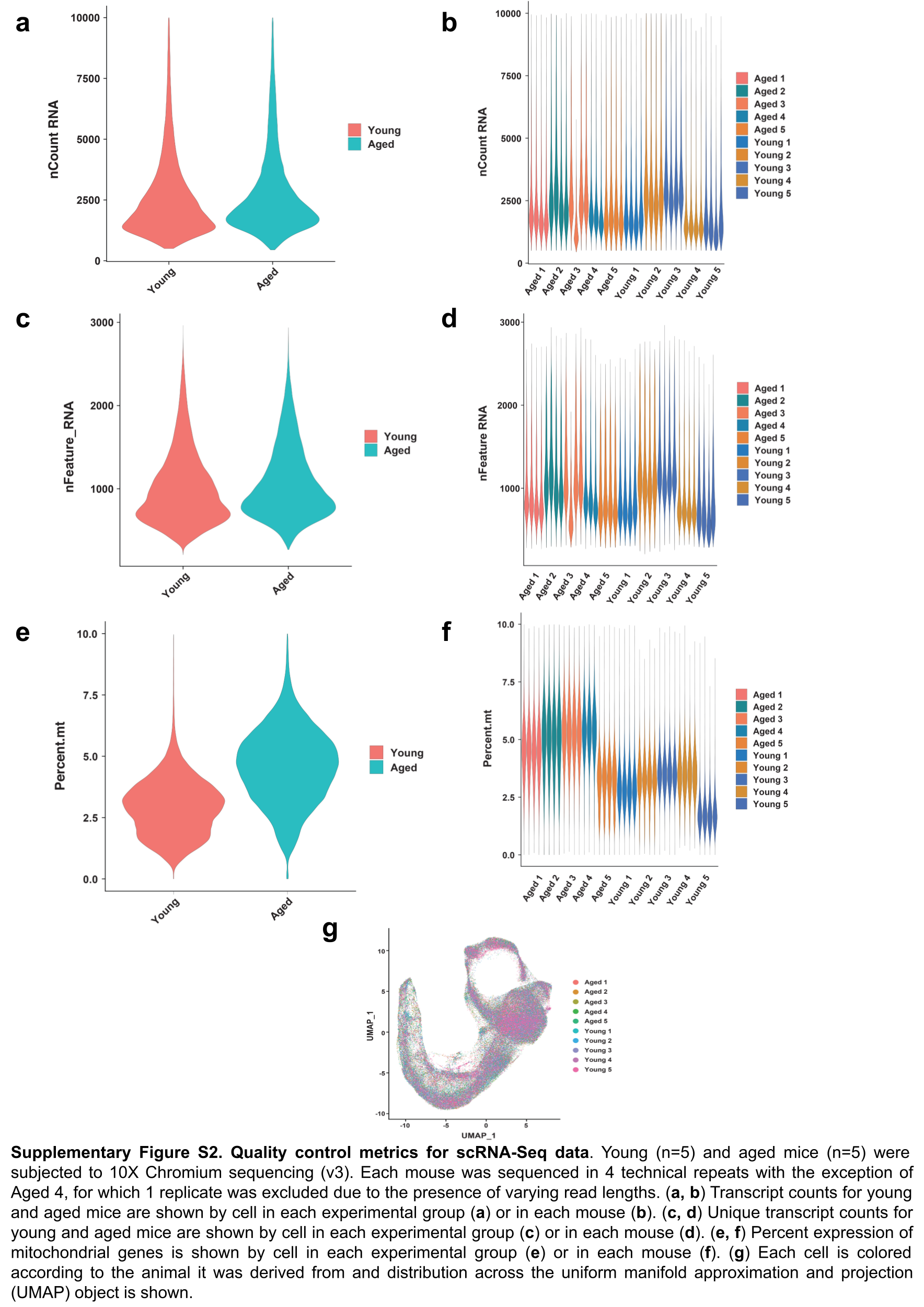
**


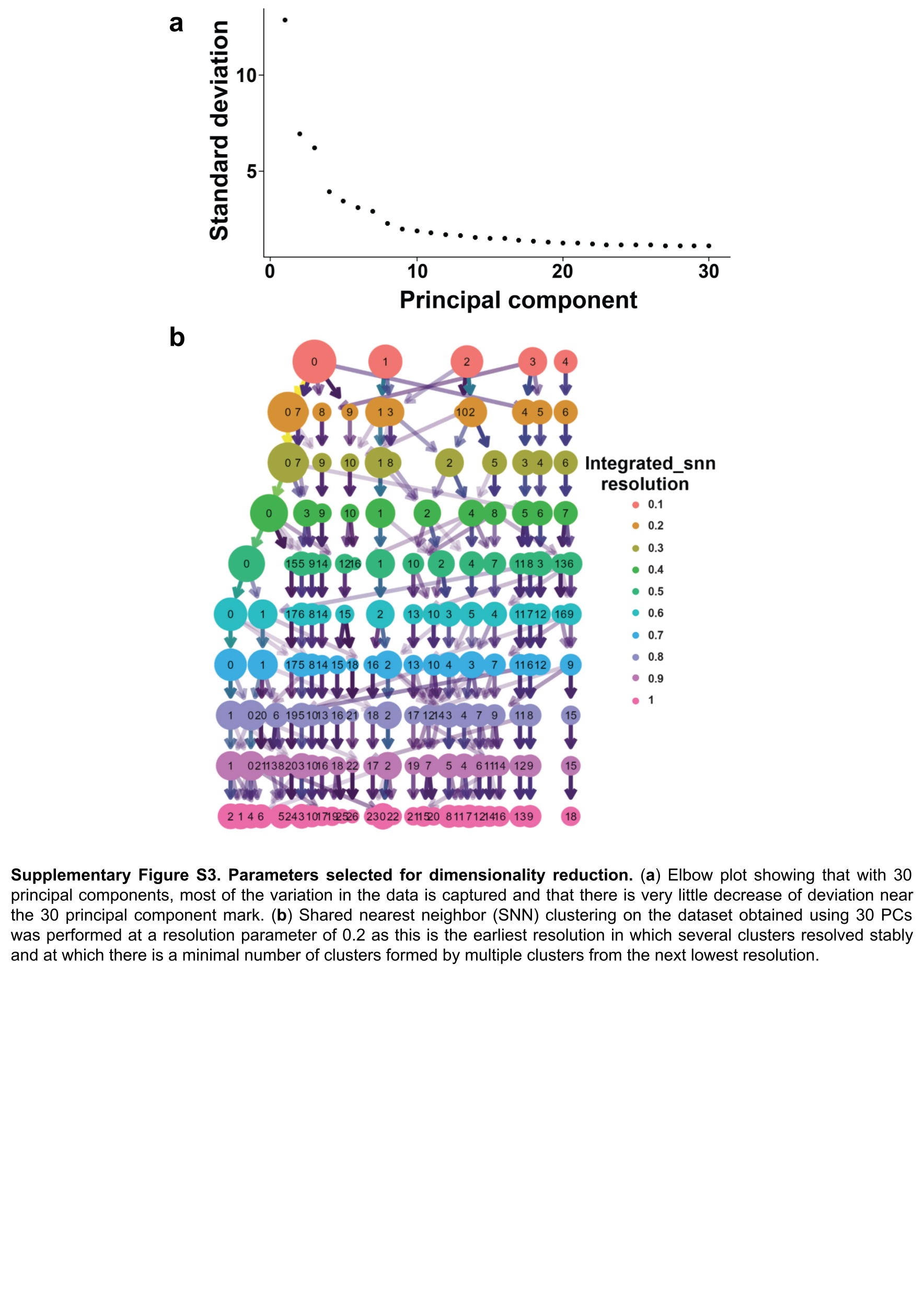


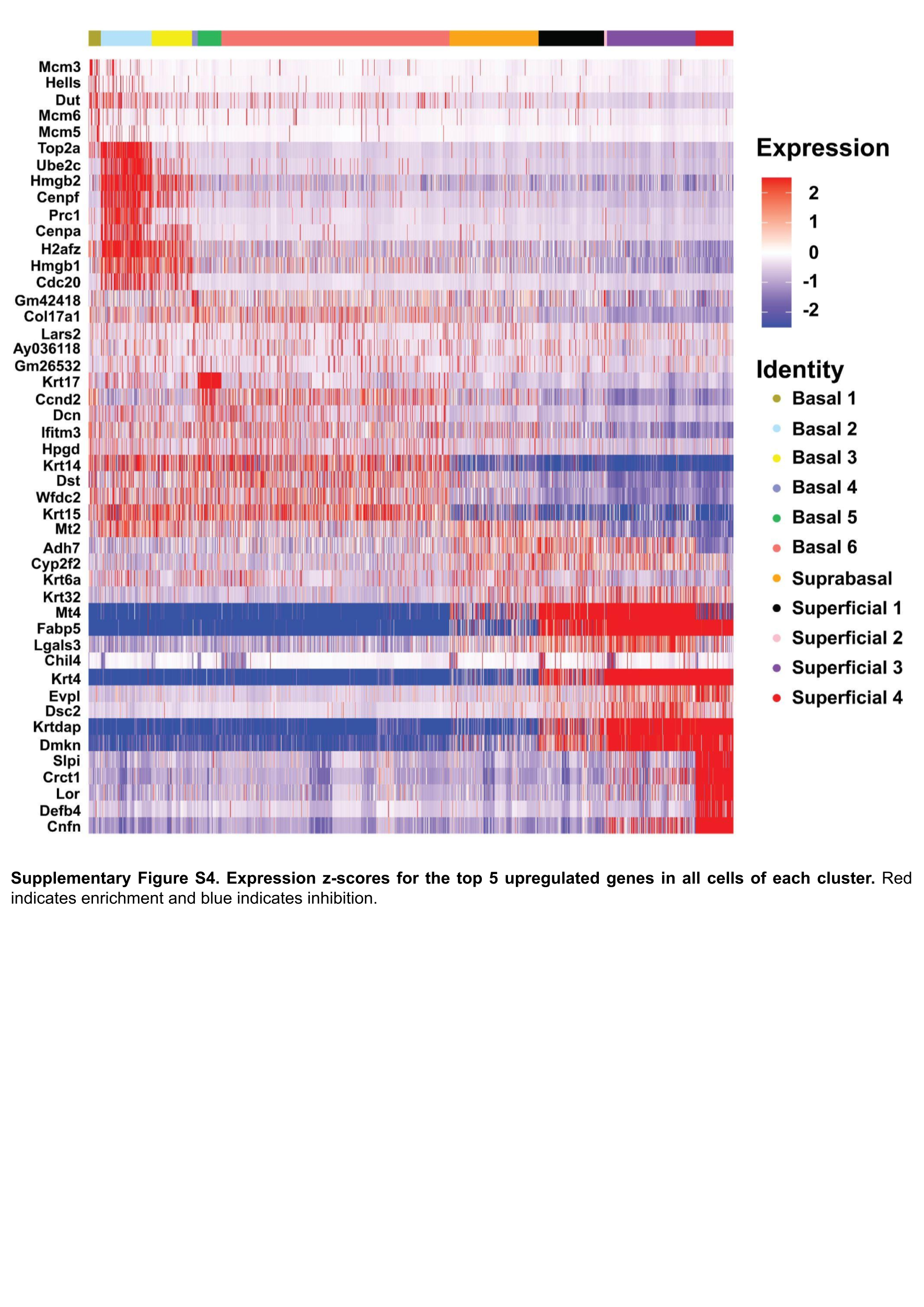


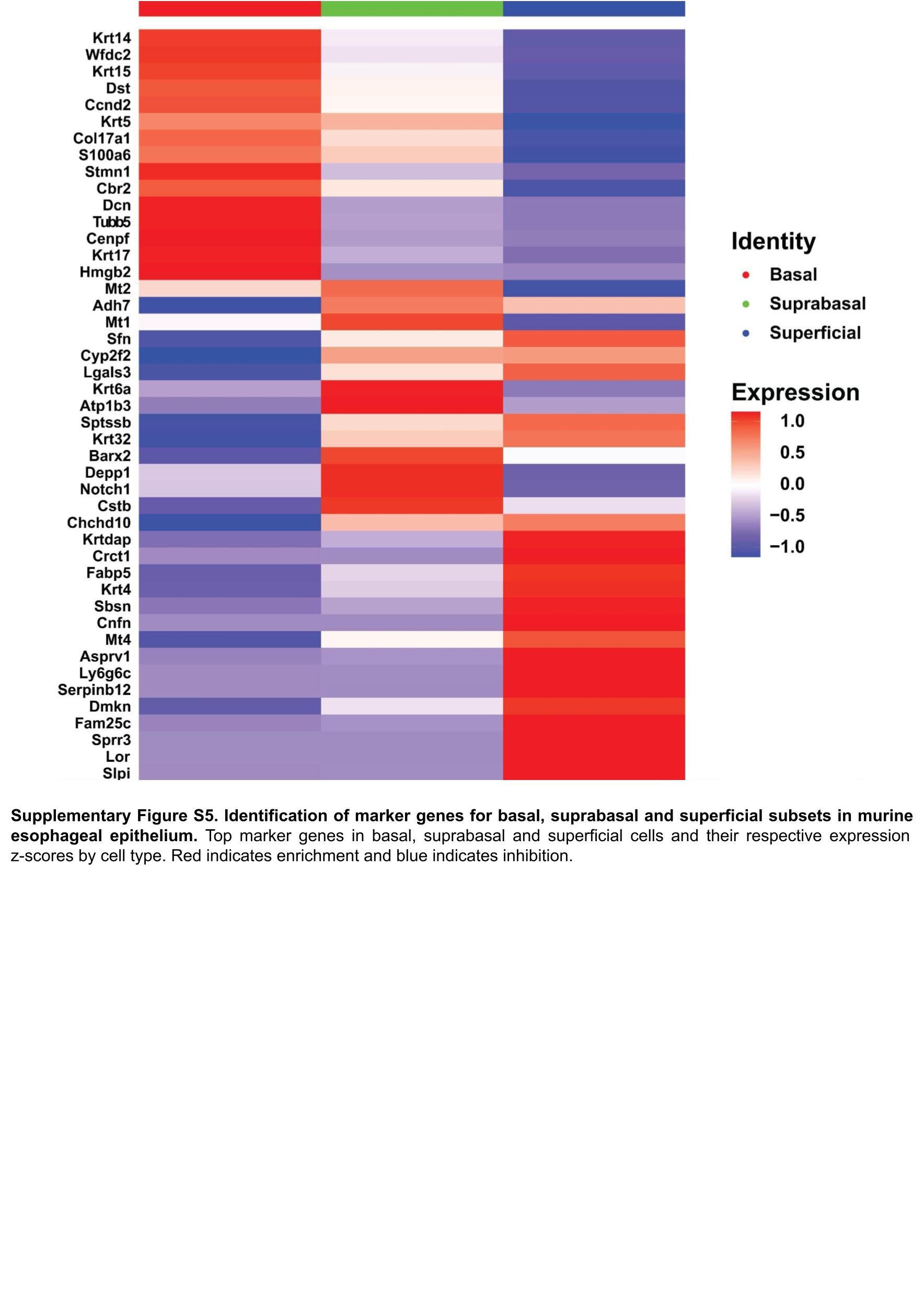


**
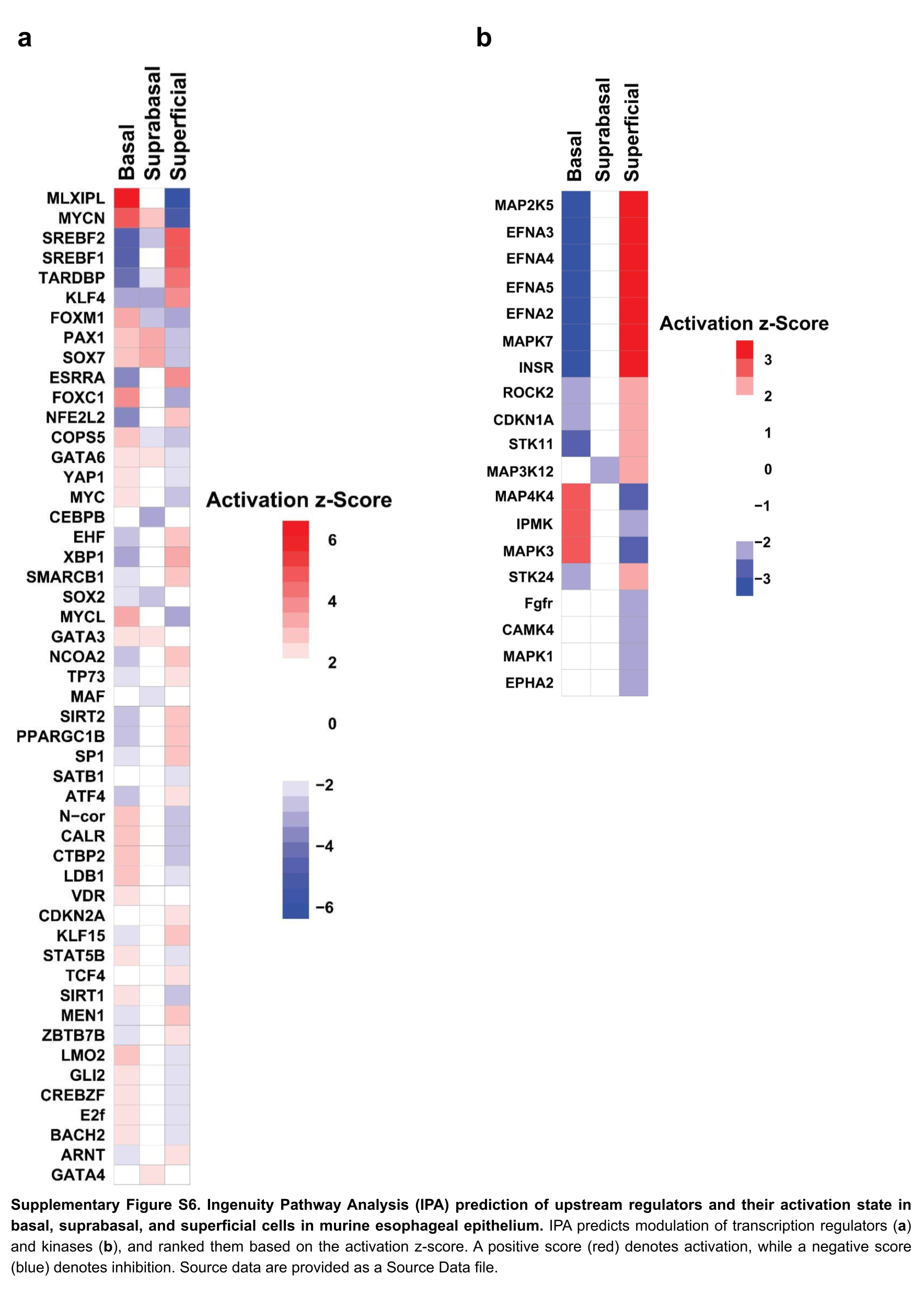
**

**
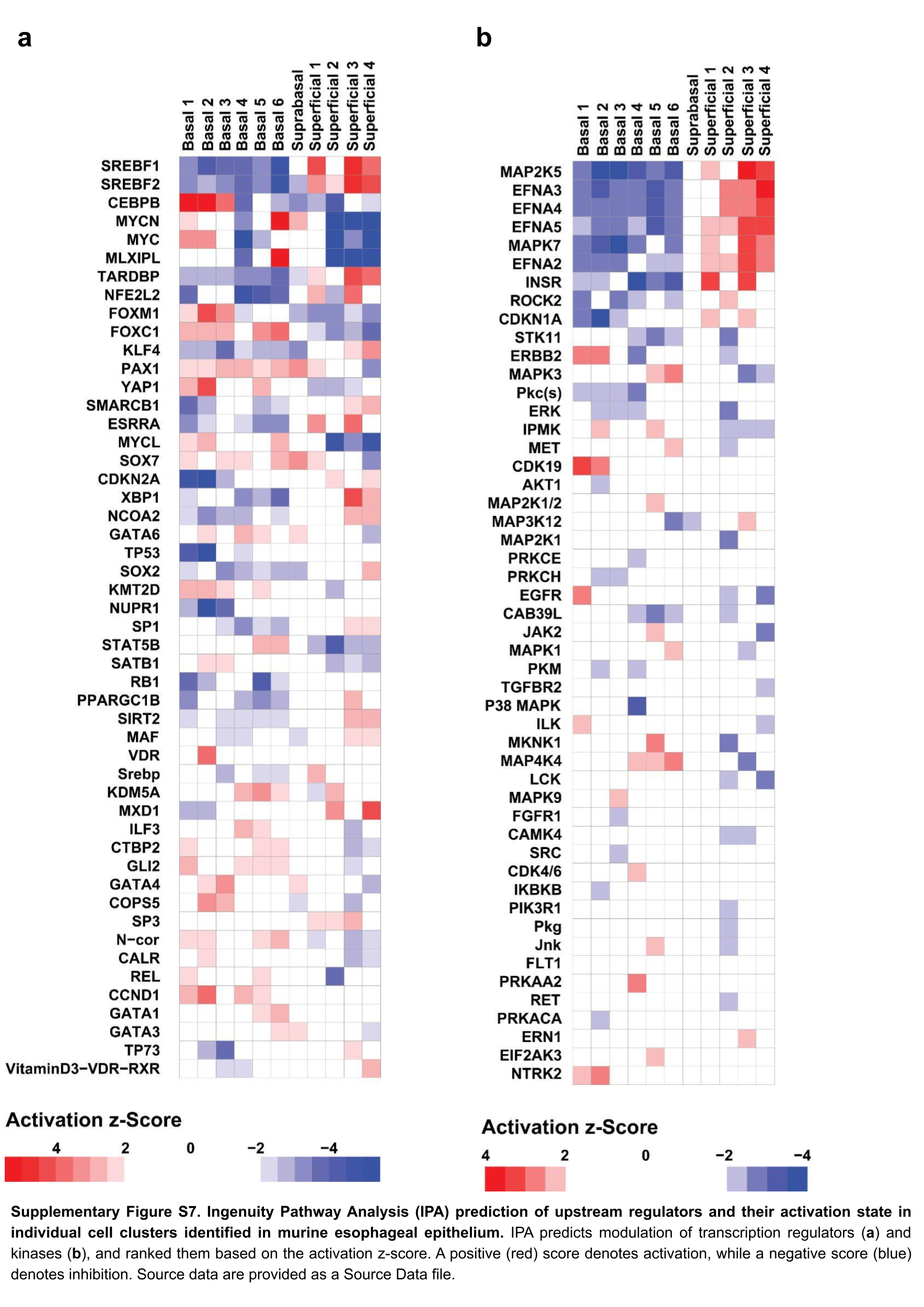
**

**
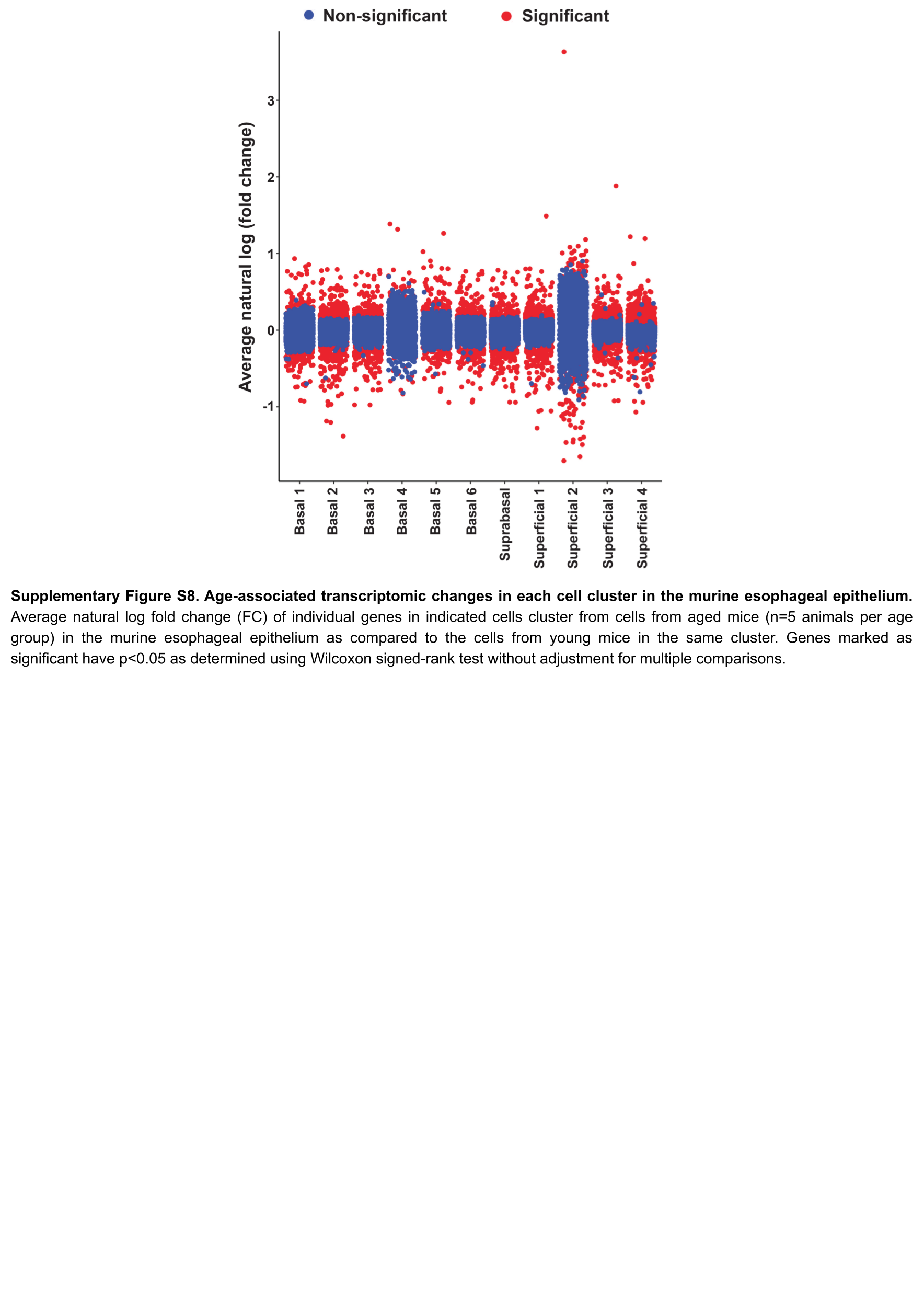
**

**
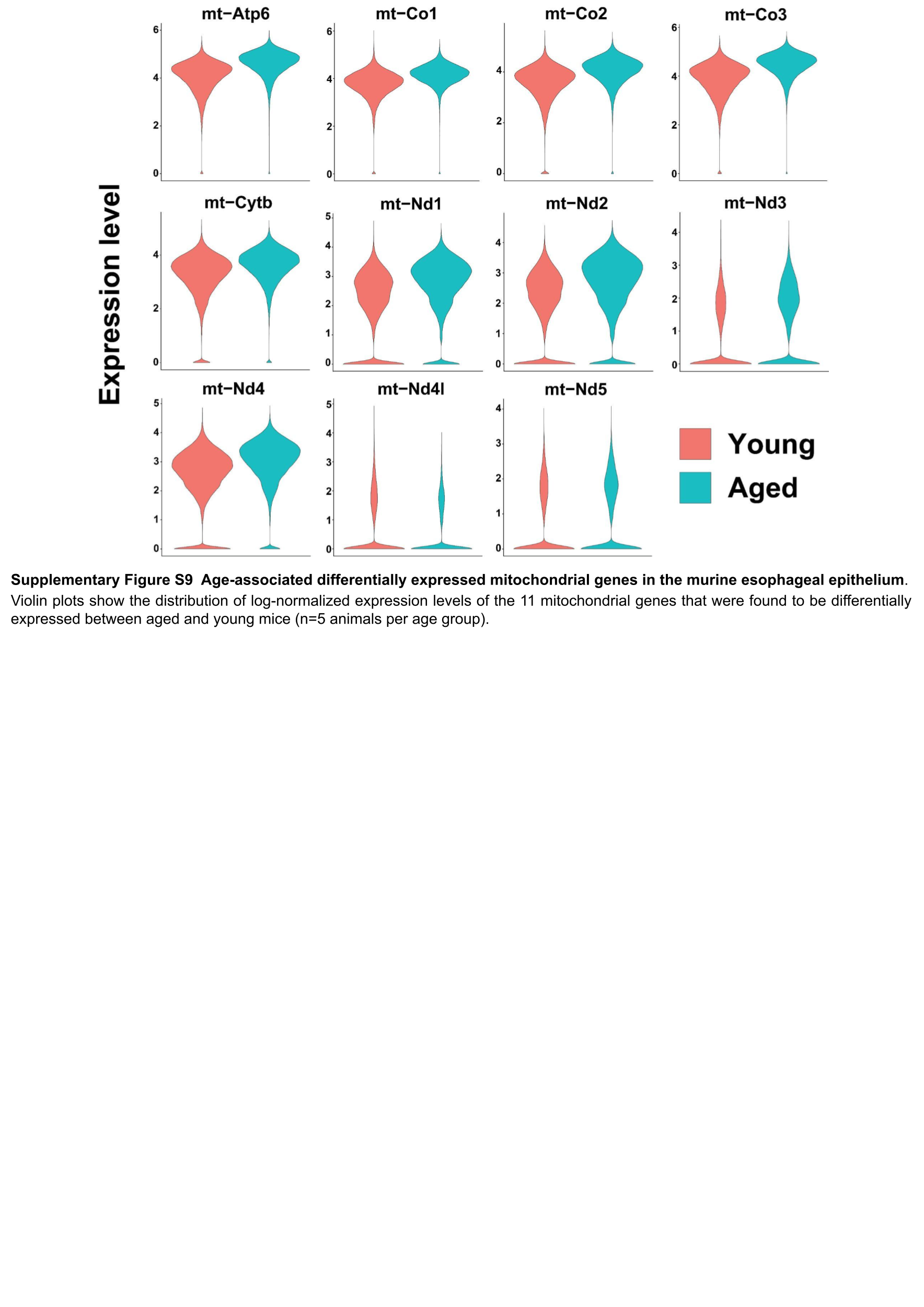
**

**
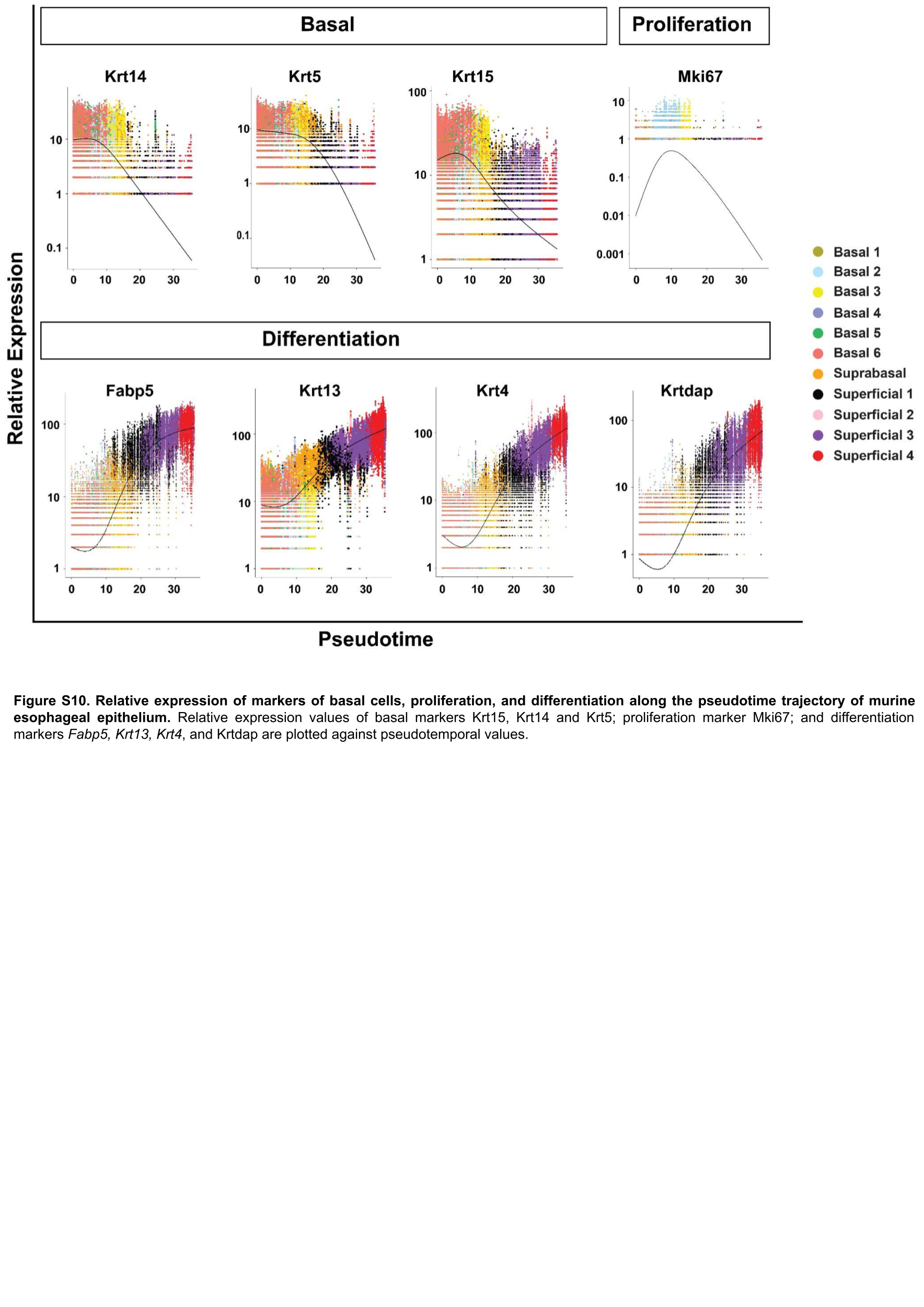
 Supplementary Table S1: Antibodies used in immunoblotting**

| **Antibody** | **Catalogue#** | **Manufacturer** | **Host species** | **Dilution** |
| --- | --- | --- | --- | --- |
| KRT5 | MA5-16372 | Invitrogen | Rabbit | 1:1000 |
| EIF2α | 9722S | Cell Signaling Technology | Rabbit | 1:1000 |
| EIF2Bε | 3595S | Cell Signaling Technology | Rabbit | 1:1000 |
| RPL10 | 72912S | Cell Signaling Technology | Rabbit | 1:1000 |
| RPS3 | 9538S | Cell Signaling Technology | Rabbit | 1:1000 |
| KRT13 | 10164-2-AP | Proteintech | Rabbit | 1:1000 |
| GSTP1 | 15902-1-AP | Proteintech | Rabbit | 1:1000 |
| GSTA4 | 17271-1-AP | Proteintech | Rabbit | 1:1000 |
| β-Actin | MA1-744 | Invitrogen | Mouse | 1:5000 |
| Anti-Rabbit IgG (H+L) HRP | 31466 | Invitrogen | Goat | 1:3000 |
| Anti-Mouse IgG (H+L) HRP | 20-304 | Genesee Scientific | Goat | 1:3000 |
