# Supplementary material for: Single cell transcriptomic analysis reveals cellular diversity of murine esophageal epithelium
Source: Nat Commun. 2022 Apr 20;13:2167. doi: 10.1038/s41467-022-29747-x (PMC9021266; doi:10.1038/s41467-022-29747-x)
Supplement: Supplementary file 2 — Supplementary Information Inventory [file 41467_2022_29747_MOESM2_ESM.docx]

**Inventory of Supporting Information**

Supplementary Information: Supplementary Figures and Legends

Supplementary Dataset 1: Source Data File
