# Supplementary material for: Single cell transcriptomic analysis reveals cellular diversity of murine esophageal epithelium
Source: Nat Commun. 2022 Apr 20;13:2167. doi: 10.1038/s41467-022-29747-x (PMC9021266; doi:10.1038/s41467-022-29747-x)
Supplement: Supplementary file 5 — Reporting Summary [file 41467_2022_29747_MOESM5_ESM.pdf]

nature portfolio

Corresponding author(s): Kelly Whelan

Last updated by author(s): Mar 7, 2022

# Reporting Summary

Nature Portfolio wishes to improve the reproducibility of the work that we publish. This form provides structure for consistency and transparency in reporting. For further information on Nature Portfolio policies, see our Editorial Policies and the Editorial Policy Checklist.

## Statistics

For all statistical analyses, confirm that the following items are present in the figure legend, table legend, main text, or Methods section.

| n/a | Confirmed | |
|---|---|---|
| ☐ | ☒ | The exact sample size ($n$) for each experimental group/condition, given as a discrete number and unit of measurement |
| ☐ | ☒ | A statement on whether measurements were taken from distinct samples or whether the same sample was measured repeatedly |
| ☐ | ☒ | The statistical test(s) used AND whether they are one- or two-sided<br>*Only common tests should be described solely by name; describe more complex techniques in the Methods section.* |
| ☐ | ☒ | A description of all covariates tested |
| ☐ | ☒ | A description of any assumptions or corrections, such as tests of normality and adjustment for multiple comparisons |
| ☐ | ☒ | A full description of the statistical parameters including central tendency (e.g. means) or other basic estimates (e.g. regression coefficient) AND variation (e.g. standard deviation) or associated estimates of uncertainty (e.g. confidence intervals) |
| ☐ | ☒ | For null hypothesis testing, the test statistic (e.g. $F$, $t$, $r$) with confidence intervals, effect sizes, degrees of freedom and $P$ value noted<br>*Give P values as exact values whenever suitable.* |
| ☒ | ☐ | For Bayesian analysis, information on the choice of priors and Markov chain Monte Carlo settings |
| ☐ | ☒ | For hierarchical and complex designs, identification of the appropriate level for tests and full reporting of outcomes |
| ☒ | ☐ | Estimates of effect sizes (e.g. Cohen's $d$, Pearson's $r$), indicating how they were calculated |

*Our web collection on statistics for biologists contains articles on many of the points above.*

## Software and code

Policy information about availability of computer code

| | |
|---|---|
| Data collection | Cell Ranger 6 |
| Data analysis | Seurat 4.0; Monocle 3.1; ggpubr 0.4; ggplot2 3.3.5; Clustree 0.4.4. on Custom scripts are available at https://github.com/alkarami/Whelan-scRNA-Esophagus-Dec21. |

For manuscripts utilizing custom algorithms or software that are central to the research but not yet described in published literature, software must be made available to editors and reviewers. We strongly encourage code deposition in a community repository (e.g. GitHub). See the Nature Portfolio guidelines for submitting code & software for further information.

## Data

Policy information about availability of data

All manuscripts must include a data availability statement. This statement should provide the following information, where applicable:

- Accession codes, unique identifiers, or web links for publicly available datasets
- A description of any restrictions on data availability
- For clinical datasets or third party data, please ensure that the statement adheres to our policy

The authors declare that all data supporting the findings of this study are available within the article and its supplementary information files. Source data are provided with this paper for the following figures: 2c, d; 3a; 4b-e; 5e; 6d; 7e; S6; S7. Gene expression and pathway information for each cluster generated in this study are provided in the Supplementary Information/Source Data file. The processed cell and gene matrices are available at GEO accession GSE193376 [https://www.ncbi.nlm.nih.gov/geo/query/acc.cgi?acc=GSE193376] as supplementary files. The repository Whelan-scRNA-Esophagus-Dec21 is archived on Zenodo under the DOI 10.5281/zenodo.6286725 on February 25, 2022.

# Field-specific reporting

Please select the one below that is the best fit for your research. If you are not sure, read the appropriate sections before making your selection.

☒ Life sciences ☐ Behavioural & social sciences ☐ Ecological, evolutionary & environmental sciences

For a reference copy of the document with all sections, see nature.com/documents/nr-reporting-summary-flat.pdf

# Life sciences study design

All studies must disclose on these points even when the disclosure is negative.

| | |
|---|---|
| Sample size | Sample size was determined by performing power calculations on published or preliminary data. |
| Data exclusions | We excluded single cell RNA-seq data for non-epithelial cell clusters (immune cells, fibroblasts, endothelial cells). This was part of the study design as the goal of the current study was to extensively characterize esophageal epithelium. Each mouse was sequenced in 4 technical replicates for single cell RNA-Seq. All replicates were used for the current study with the exception of a single replicate from Aged mouse 4 due to the presence of varying read lengths. |
| Replication | All experiments were performed in at least 3 independent animals (in vivo and in situ studies) or 3 independent trials (in vitro studies) |
| Randomization | Mice were purchased from Jackson labs at the appropriate age for all experiments. No randomization was necessary. |
| Blinding | Personnel were blinded to experimental conditions during data collection and analysis. |

# Reporting for specific materials, systems and methods

We require information from authors about some types of materials, experimental systems and methods used in many studies. Here, indicate whether each material, system or method listed is relevant to your study. If you are not sure if a list item applies to your research, read the appropriate section before selecting a response.

## Materials & experimental systems

| n/a | Involved in the study | |
|---|---|---|
| ☐ | ☒ | Antibodies |
| ☒ | ☐ | Eukaryotic cell lines |
| ☒ | ☐ | Palaeontology and archaeology |
| ☐ | ☒ | Animals and other organisms |
| ☒ | ☐ | Human research participants |
| ☒ | ☐ | Clinical data |
| ☒ | ☐ | Dual use research of concern |

## Methods

| n/a | Involved in the study | |
|---|---|---|
| ☒ | ☐ | ChIP-seq |
| ☒ | ☐ | Flow cytometry |
| ☒ | ☐ | MRI-based neuroimaging |

## Antibodies

| | |
|---|---|
| Antibodies used | KRT5 (MA5-16372; Invitrogen; 1:1000), EIF2A (9722S; Cell Signaling Technology; 1:1000), EIF2BE (3595S; ell Signaling Technology; 1:1000), RPL1:100 (2912S; Cell Signaling Technology; 1:1000), RPS3 (9538S; Cell Signaling Technology; 1:1000), KRT13 (10164-2-AP, Proteintech; 1:1000) ; GSTP1 (15902-1-AP; Proteintech; 1:1000), GSTA4 (17271-1-AP; Proteintech; 1:1000); B-Actin (MA1-744; Invitrogen:1;5000), Anti-Rabbit IgG (H+L) HRP (31466; Invitrogen; 1:3000); Anti-Mouse IgG (H+L) HRP (20-304; Invitrogen; 1:3000), COL17A1 (Invitrogen, MA5-24848l, Clone 2C3 1:100), ATP1B3 (Abcam, ab137055; Clone EPR8981, 1:100) and CNFN (Novus Biologicals, NBP2-14668; 1:100). |
| Validation | Each antibody used for IHC was validated in murine esophageal epithelium using a no primary antibody control. |

## Animals and other organisms

Policy information about studies involving animals; ARRIVE guidelines recommended for reporting animal research

| | |
|---|---|
| Laboratory animals | Both male and female wild type C57Black6/6 mice (Cat# 000664) were purchased from Jackson Laboratories at age 10 weeks or 70 weeks. Mice were allowed to acclimate for at least 2 weeks prior to use for experiments. Information on housing conditions in provided in the Methods. |
| Wild animals | *Provide details on animals observed in or captured in the field; report species, sex and age where possible. Describe how animals were caught and transported and what happened to captive animals after the study (if killed, explain why and describe method; if released, say where and when) OR state that the study did not involve wild animals.* |

Field-collected samples

*For laboratory work with field-collected samples, describe all relevant parameters such as housing, maintenance, temperature, photoperiod and end-of-experiment protocol OR state that the study did not involve samples collected from the field.*

Ethics oversight

All studies were completed under a Temple University Institutional Animal Care & Use Committee-approved protocol (#5018). This in noted in the Methods.

Note that full information on the approval of the study protocol must also be provided in the manuscript.
